# Zooming in on Butyrate-Producing Clostridial Consortia in the Fermented Grains of *Baijiu via* Gene Sequence-Guided Microbial Isolation

**DOI:** 10.3389/fmicb.2019.01397

**Published:** 2019-06-21

**Authors:** Li-Juan Chai, Zhen-Ming Lu, Xiao-Juan Zhang, Jian Ma, Peng-Xiang Xu, Wei Qian, Chen Xiao, Song-Tao Wang, Cai-Hong Shen, Jin-Song Shi, Xu Zheng-Hong

**Affiliations:** ^1^ National Engineering Laboratory for Cereal Fermentation Technology, Jiangnan University, Wuxi, China; ^2^ Key Laboratory of Industrial Biotechnology of Ministry of Education, School of Biotechnology, Jiangnan University, Wuxi, China; ^3^ School of Pharmaceutical Science, Jiangnan University, Wuxi, China; ^4^ Jiangsu Engineering Research Center for Bioactive Products Processing Technology, Jiangnan University, Wuxi, China; ^5^ National Engineering Research Center of Solid-State Brewing, Luzhou, China

**Keywords:** butyric acid, *baijiu* fermented grains, complex microbial community, Clostridia, *Clostridium* isolation

## Abstract

Butyrate, one of the key aroma compounds in Luzhou-flavor *baijiu*, is synthesized through two alternative pathways: butyrate kinase (*buk*) and butyryl-CoA: acetate CoA-transferase (*but*). A lack of knowledge of butyrate-producing microorganisms hinders our ability to understand the flavor formation mechanism of *baijiu*. Here, temporal dynamics of microbial metabolic profiling in fermented grains (FG) was explored *via* PICRUSt based on 16S rRNA gene sequences. We found Bacilli and Bacteroidia were the major potential butyrate producers in *buk* pathway at the beginning of fermentation, while later Clostridia dominated the two pathways. Clone library analysis also revealed that Clostridia (~73% OTUs) was predominant in *buk* pathway throughout fermentation, followed by Bacilli and Bacteroidia, and *but* pathway was merely possessed by Clostridia. Afterward, Clostridia-specific 16S rRNA gene sequencing demonstrated *Clostridium* might be the major butyrate-producing genus in two pathways, which was subsequently evaluated using culture approach. Seventeen *Clostridium* species were isolated from FG based on 16S rRNA gene sequence-guided medium prediction method. Profiles of short-chain fatty acids and *but* and *buk* genes in these species demonstrated phylogenetic and functional diversities of butyrate-producing *Clostridium* in FG. These findings add to illustrate the diversity of potential butyrate producers during brewing and provide a workflow for targeting functional microbes in complex microbial community.

## Introduction

Luzhou-flavor *baijiu* is a kind of Chinese distilled spirits produced from different grains (mainly sorghum) by spontaneous solid-state fermentation in a mud pit, and it accounts for more than 70% of Chinese *baijiu* production (Tao et al., 2014). The production process of Luzhou-flavor *baijiu* can be generally divided into three phases: Daqu (fermentation starter) making, in-cellar fermentation, and distillation (Zheng and Han, 2016). During the in-cellar fermentation stage, Daqu, steamed grains, and rice husk are mixed and sealed in a fermentation pit for around 40–60 days. The mixed grains in fermentation pit provide the habitat and substrate for the proliferation and metabolism of microorganisms during brewing (Wang et al., 2008). A variety of flavor compounds are formed in fermented grains through the metabolism of multiple brewing microorganisms (Wang et al., 2014), determining the typical and unique flavor of Luzhou-flavor *baijiu* (Fang et al., 2019), although it is mainly constituted of ethanol (38–65%, v/v) and water (Zheng and Han, 2016). The representative aroma compounds of Luzhou-flavor *baijiu* are predominantly ethyl hexanoate, ethyl butanoate, ethyl acetate, ethyl lactate and their corresponding acids, hexanoic acid, butanoic acid, acetic acid, and lactic acid (Fan and Qian, 2006; Liu and Sun, 2018; Fang et al., 2019). The aroma intensity characterized by Osme values of butanoic acid (rancid and cheesy aroma, Osme value = 15) and its main derivative ethyl butanoate (pineapple aroma, Osme value ≥14) almost ranked first among the volatile compounds according to Fan and Qian (2006). Thus, shedding light on the butyrate-producing microbial consortia in fermented grains is conductive to elucidate the flavor formation mechanism of Luzhou-flavor *baijiu*.

Culture-independent approaches (e.g., DGGE, 16S rRNA sequencing) revealed that Bacilli, Bacteroidetes, and Clostridia dominated the bacterial consortia in fermentation grains (Wang et al., 2008), either during brewing (Wang et al., 2017) or from different-aged fermentation cellars (Ding et al., 2015). At the genus level, *Lactobacillus* was predominant, particularly at the later stage of fermentation (Wang et al., 2017). Using culture method, most of the isolated bacteria were scattered in *Bacillus*, *Lactobacillus*, and *Acetobacter* (Zou et al., 2018b). By performing transcriptome analysis, synergistic effect was identified between *Lactobacillus* and *Saccharomyces* in the sulfur compound production (Liu et al., 2017). Moreover, *Lactobacillus* and *Bacillus* were found as dominant producers of lactic acid and other organic acids. However, as one of the key aroma compounds in Luzhou-flavor *baijiu*, taxonomic distribution of butyrate-producing microorganisms in fermentation grains during brewing remains unclear to date.

**Figure 1:**
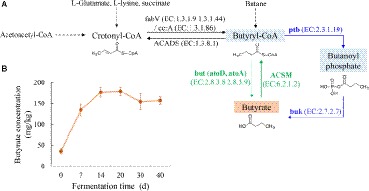
**(A)** Putative microbial butyrate metabolism pathway in the fermented grains of Luzhou-flavor *baijiu* based on the information from KEGG database (ko00650). The enzyme IDs in KEGG were shown in brackets. Key enzymes involved in *but* / *buk* pathway were highlighted by green/blue color. **(B)** Butyrate content in fermented grains during brewing.

For the final step of microbial butyrate synthesis from butyryl-CoA, there are two alternative pathways namely butyrate kinase (*buk*) pathway and butyryl-CoA:acetate CoA-transferase (*but*) pathway (Figure 1A). The key genes *but* and *buk* were previously chosen to identify these two pathways (Louis et al., 2010; Vital et al., 2014, 2015). In anaerobic environments, butyrate could be synthesized by butyrate-producing bacteria related to *Eubacterium* spp. and *Roseburia* spp. (*Clostridium* cluster XIVa) and *Faecalibacterium prausnitzii* (*Clostridium* cluster IV) (Louis and Flint, 2009, 2017). The fermentation of Luzhou-flavor *baijiu* was also carried out under an anaerobic environment. Metagenomic analysis showed that *Clostridium* was most likely involved in butyrate production through *buk* pathway in pit mud (Tao et al., 2017). However, most of Clostridial microbes are difficult to be isolated and cultivated owing to their oxygen sensitivity. Thus, obtaining natural strains with high-yielding butyrate potential is challenging for researchers.

In the previous work, Clostridia class was identified as the predominant putative butyrate producers in both *buk* and *but* pathways in pit mud (i.e., fermentation container) of Luzhou-flavor *baijiu* and the butyrate-producing potential of some *Clostridium* strains were evaluated (Chai et al., 2019). To further unravel the source of butyrate in *baijiu* brewing ecosystem, the present study aimed to investigate the butyrate-producing bacterial consortia in *baijiu* fermented grains (i.e., fermentation substrate). Temporal distribution of functional genes involved in the conversion between butyryl-CoA and butyrate was firstly illustrated. The potential butyrate-producers were further investigated *via* the analysis of *buk* and *but* genes’ phylogenetic diversities. Subsequently, the phylogenetic diversity and pathway distribution of the major butyrate-producing group, Clostridia, was conducted. The major potential butyrate-producing *Clostridium* was isolated and their butyrate-producing capacity was assessed. This work is expected to elucidate the main potential butyrate-producing bacterial community in fermented grains during *baijiu* brewing.

## Materials and Methods

### Sampling and Storage

Fermented grains used in this study were collected from the fermentation pit of 30 years old (length × width × height = 4.3 m × 3.3 m × 2.3 m) in Luzhou Laojiao Group Co., Ltd. (Sichuan, China) (Supplementary Figure S1). Triplicate samples were obtained from three different points near the pit center on days 0, 7, 14, 20, 30, and 40. For isolation of microorganisms, the samples were stored at 4°C. For physiochemical properties analysis, the samples were stored at −20°C. For DNA extraction, the samples were pulverized homogeneously in liquid nitrogen and stored at −80°C.

### Measurement of Physiochemical Properties

Two grams of fermented grains were suspended in 20 ml sterile distilled water, soaked by rotational shaking at 100 rpm for 3 h at room temperature and then centrifuged at 10,000× *g* for 15 min. The supernatant extract was collected for butyric acid and lactic acid analysis, which were determined by high performance liquid chromatography (HPLC) method as follows. The clear supernatant (5 ml) was mixed thoroughly with 2 ml zinc sulfate (300 g/L) and 2 ml potassium ferrocyanide (106 g/L). The mixture was centrifuged at 10,000× *g* for 10 min, followed by filtration through a 0.22 μm nylon syringe filter (ALWSCI, Zhejiang, China). A Waters E2695 HPLC system with a 2998 PDA detector was used for two kinds of acids assessment. Determination of lactic acid was performed using a Waters Atlantis T3 column (5 μm, 4.6 mm × 250 mm) with 30°C column temperature and 20 mM NaH_2_PO_4_ as the mobile phase (pH 2.7, 0.7 ml/min). As for butyric acid analysis, the SEPAX Carbomix H-NP 5:8 column (5 μm, 7.8 mm × 300 mm) temperature was maintained at 55°C and the flow of mobile phase (2.5 mM H_2_SO_4_) was 0.6 ml/min. For both of them, the injection amount was 10 μl and detection wavelength was 210 nm.

Temperature during fermentation process was measured with electronic temperature probes (PXTONG, PX-12). Ethanol content of fermented grains was detected according to the protocol described by Shen (2011). pH was detected using a Mettler Toledo FiveEasy Plus™ pH/mV meter equipped with an LE438 solid electrode (Mettler Toledo Instruments, Shanghai, China). Moisture content of fermented grains was determined immediately after sampling through a gravimetric approach by drying at 105°C for 4 h in the oven. All experiments were performed in three replicates.

### DNA Extraction and PCR Amplification

DNA was extracted from fermented grains using the PowerSoil^®^ DNA Isolation Kit (MOBIO Laboratories, Inc., Carlsbad, CA, USA) following the manufacturer’s protocol. For each reaction, 0.5 g pulverized powder was used. The quality and quantity of DNA extracted was measured using a NanoDrop 3300 spectrophotometer (Wilmington, USA).

All primers used in this study are shown in Supplementary Table S1. Degenerate primers for *buk* and *but* genes were designed *via* the CODEHOP method^1^ (Rose et al., 2003; Chai et al., 2019). The *but* and *buk* genes were amplified as described by Vital et al. (2013) with some modifications. The PCR programs consisted of an initial 5 min denaturation at 94°C, followed by 35 cycles of denaturation at 94°C for 30 s, annealing at 47°C (*buk*) or 49°C (*but*) for 30 s, extension at 72°C for 30 s, and a final elongation step of 5 min at 72°C.

With regard to bacterial consortia analysis in fermented grains, the V1/V3 hypervariable region (*Escherichia coli* positions 5-534) of 16S rRNA gene was amplified with the P1/P2 primer set (Huang et al., 2011). The Clostridia-specific primer set SJ-F/SJ-R targeting V4/V5 hypervariable region (*E. coli* positions 679-952) of 16S rRNA gene was used to specifically unravel the Clostridial community in fermented grains (Hu et al., 2014). The detailed PCR programs of these two primer sets were described in previous reports. At the 5′ end of each primer set used for high-throughput sequencing, unique multi-nucleotide sequences (18 bases) were synthesized as barcodes to assign sequences to different samples. The bacterial 27F/1492R primer set was used to amplify nearly full length 16S rRNA gene of the isolated strains (Zhang et al., 2014), and the PCR program was as follows: one cycle at 95°C for 5 min, followed by 30 cycles at 95°C for 60 s, 55°C for 30 s and 72°C for 90 s, and a final extension at 72°C for 5 min.

### Processing and Analyzing Bacterial 16S rRNA Gene Amplicon Sequencing Data

To characterize the bacterial community in fermented grains, equivalent concentration of the barcoded 16S rRNA gene amplicons targeting V1/V3 hypervariable region were pooled and sequenced using a Roche/454 Genome Sequencer FLX Titanium. Mean length of the produced reads was 400 bp. The sequencing data was processed and analyzed *via* MOTHUR (version 1.35.1) according to the previous report (Xiao et al., 2017). To generate clean reads, reads shorter than 150 bp were discarded and reads with average quality score <20 or unknown nucleotides or any homopolymers of more than eight bases or without the primer sequence were removed from the raw data. Chimeric sequences identified by UCHIME were discarded as well (Edgar et al., 2011). Afterward, high-quality reads were clustered into individual OTUs at a 97% identity threshold *via* UPARSE^2^ (Edgar, 2013). Afterward, the representative sequence from each OTU was aligned against the RDP^3^ and Greengenes (version gg_13_5) databases at a minimum of 80% identity for taxonomic annotation. OTUs that were not classified to the genus level were aligned to the EzBioCloud database (Yoon et al., 2017). The basic sequencing information and α-diversity indices were summarized in Supplementary Table S2.

The KEGG Ortholog (KO) functional profiles of microbial communities were predicted based on 16S rRNA gene sequences *via* the PICRUSt approach (Phylogenetic Investigation of Communities by Reconstruction of Unobserved States), which was performed following the workflow in Galaxy web^4^ under default parameter values (Langille et al., 2013). Referring to the butanoate metabolism pathway (ko00650), taxonomic distributions of potential butyrate-producing microbes in fermented grains were analyzed depending on buk (butyrate kinase, EC 2.7.2.7) and ptb (phosphate butyryltransferase, EC 2.3.1.19) in *buk* pathway; and atoD (acetate CoA/acetoacetate CoA-transferase alpha subunit, EC 2.8.3.8 2.8.3.9), atoA (acetate CoA/acetoacetate CoA-transferase beta subunit, EC 2.8.3.8 2.8.3.9) and ACSM (medium-chain acyl-CoA synthetase, EC 6.2.1.2) in *but* pathway. Then, Spearman’s rank correlations between the predicted butyrate producers’ succession and physiochemical properties dynamics during the fermentation process of fermented grains were assessed by SPSS (version 20.0) based on the squared Euclidean distance. Correlation coefficient *r* ≥ 0.8 or ≤ −0.8 and *p* <0.05 were set as cutoffs to evaluate the probability and significance.

### Clone Library Analysis of *but* and *buk* Partial Gene Amplicons

Clone libraries based on *buk* and *but* partial gene amplicons were constructed to investigate the microorganisms with butyrate-producing potential. The PCR reaction was performed with ExTaq DNA polymerase (TAKARA, Dalian, China) with a total volume of 25 μl and 20 ng DNA from a mixture of each sample as template. The target PCR products located around 500 bp (*buk*) and 450 bp (*but*) were purified using GK 2043-200 gel extraction kit (GENERAY, Shanghai, China). Then enriched amplicons were ligated with pMD19-T vector (TAKARA, Dalian, China) and transformed into *E. coli* JM109 competent cells. At least 100 recombinant clones were randomly picked for each gene and sent to Sangon Biotech (Shanghai, China) for sequencing. Nucleotide sequences were firstly trimmed in order to avoid the influences of degenerate bases and then clustered into operational taxonomic units (OTUs) under the threshold of 95% identity by Fungene Pipeline^5^. The OTUs were aligned against the UniProtKB database^6^ through BLASTx to determine their functional annotation and taxonomic classification. Their phylogenetic analysis based on neighbor-joining algorithm was conducted and visualized using MEGA version 7 (Kumar et al., 2016).

### Processing and Analyzing Clostridial-Specific 16S rRNA Gene Amplicon Sequencing Data

Amplicons of Clostridia-specific V4/V5 hypervariable region of 16S rRNA gene were purified using a SanPrep Column PCR Product Purification Kit (Sangon Biotech, Shanghai, China). Barcoded amplicons were pooled in equal concentrations and sequenced using Illumina MiSeq Benchtop Sequencer (2 × 300 bp). Quality-filtration and analysis of the sequencing data was performed following the standard workflow in QIIME (version 1.9.1) (Caporaso et al., 2010; Bokulich et al., 2013). The raw datasets were initially demultiplexed based on barcode and primer prior to assembly using FLASH (version 1.2.7) (Magoč and Salzberg, 2011). After quality filtration under QIIME pipeline and chimera removal using the UCHIME algorithm (Edgar et al., 2011), clean tags were clustered into OTUs at 97% similarity by UPARSE (Edgar, 2013). The representative sequences were then assigned to taxonomy with the Greengenes (version gg_13_5) and RDP classifier using 80% identity as a cutoff on QIIME. OTUs that were not classified to the genus level were aligned to the EzBioCloud database (Yoon et al., 2017). The basic sequencing information and α-diversity indices were summarized in Supplementary Table S2. The phylogenetic trees displaying the dynamic changes of Clostridial community in fermented grains during brewing were constructed using MEGAN 5.10.6 (Mitra et al., 2011). Functional composition of Clostridia in butyrate metabolism pathway during the fermentation process was predicted by PICRUSt (Langille et al., 2013).

### 16S rRNA Gene-Guided Isolation of *Clostridium* and Metabolite Analysis by HPLC

Based on the OTUs’ sequences from Clostridia-specific 16S rRNA gene sequencing, species-specific culture media of *Clostridium* were predicted by a phylogeny-based predictor, called GROWREC, in the Known Media Database (KOMODO, http://komodo.modelseed.org) under default parameters (Oberhardt et al., 2015). The *Clostridium* strains were isolated by the predicted media from the fermented grains under an anaerobic system (DG250, Don Whitely Scientific, UK), including 80% N_2_, 10% CO_2_, and 10% H_2_. DNA of the isolated strains was extracted by a Bacterial Genomic DNA Extraction Kit (DP302) (Tiangen, Beijing, China). Their 16S rRNA genes were amplified with 27F/1492R primer set (Zhang et al., 2014) and the obtained sequences were blasted against EzBioCloud database for taxonomic verification (Yoon et al., 2017). The potential butyrate-producing pathway for each verified strain was detected *via but* and *buk* genes amplification with *but*-F/*but*-R and *buk*-F/*buk*-R primer sets, respectively.

To quantify short-chain fatty acids (SCFA), the isolates were cultured in liquid reinforced clostridial medium (RCM, pH 6.5) at 37°C for 7 days in the above-mentioned anaerobic system. RCM included 10.0 g/L peptone, 10.0 g/L beef extract, 3.0 g/L yeast extract, 5.0 g/L glucose, 1.0 g/L soluble starch, 0.5 g/L cysteine hydrochloride, 3.0 g/L sodium chloride, and 3.0 g/L sodium acetate with 1.0 mg/L resazurin (a redox reaction indicator). Production of SCFA for each isolate was analyzed using the same HPLC workflow as butyrate described in section “Measurement of Physiochemical Properties.” All culture experiments were performed in three biological replicates. The 16S rRNA gene sequences of the isolates have been deposited in NCBI/GenBank database with the accession numbers MK720976-MK720991.

## Results

### Physiochemical Features of Fermented Grains Throughout Fermentation

Variability patterns of fermented grains’ physiochemical properties during brewing were recorded (Supplementary Figure S2). Temperature increased by around 10°C at the first 7 days of fermentation, afterward, decreased from 33 to 25°C till the end, changing about 2°C per 10 days. The accumulation of ethanol mainly occurred along with the temperature upregulation. Water content of fermented grains fluctuated between 53 and 57% during fermentation. Acid fermentation environment was observed by pH detection. Lactic acid was one of the major organic acids in fermented grains, which had the tendency of upregulation through fermentation. As for butyric acid content, dramatical increase happened primarily from day 0 to 14, and kept relatively stable until the end of fermentation (Figure 1B).

### Predictive Distribution of Key Enzymes Involved in Bacterial Butyrate Metabolism Pathways

**Figure 2:**
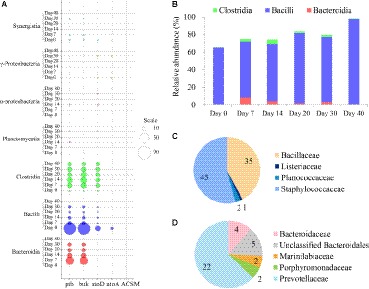
Temporal distribution of butyrate producers in the fermented grains of Luzhou-flavor *baijiu*. **(A)** Taxonomic distribution and enzymes (ptb, buk, atoD, atoA and ACSM) reads for butyrate formation in the microbial community of fermented grains *via* PICRUSt analysis. The diameters of bubbles (scale) are proportional to the read numbers of enzymes. **(B)** Succession of three major classes (Clostridia, Bacilli, and Bacteroidia) related to butyrate synthesis based on sequencing of 16S rRNA gene. **(C)** Family-level diversity of buk-associated Bacilli in the fermented grains on day 0. The numbers represent the read numbers of buk. **(D)** Family-level diversity of buk-associated Bacteroidia in the fermented grains on day 7. The numbers represent the read numbers of buk.

With respect to bacterial butyrate metabolism, enzymes ptb and buk in *buk* pathway and atoD, atoA, and ACSM in *but* pathway was considered to be directly related to the conversion between butyryl-CoA and butyrate (Figure 1A). It should be mentioned that but is named acetate CoA/acetoacetate CoA-transferase (i.e., atoD and atoA) in the KEGG Orthology system. Thus, temporal taxonomic distribution of these crucial enzymes during brewing was assessed based on 16S rRNA gene sequencing data and their metabolic profiles inferring by PICRUSt analysis. Qualified resulting reads from the amplicon sequencing of 16S rRNA gene’s V1-V3 hypervariable region were classified into 942 OTUs. Predictive functional profiling of microbial communities demonstrated *buk* pathway was the potential major butyrate biosynthesis pathway (Figure 2A). Bacilli, Bacteroidia, and Clostridia were identified as putative dominant bacteria involved in butyrate synthesis in fermented grains throughout fermentation of Luzhou-flavor *baijiu*; nevertheless, these three classes displayed divergent butyrate metabolic features.

OTUs classified as members of the Bacilli class were found to be the most abundant in fermented grains during brewing with relative abundance varying from 63.83 to 97.44% (Figure 2B). Bacilli was considered as the major potential butyrate-producing community in the fermented grains at the very beginning of fermentation (day 0). Bacillaceae and Staphylococcaceae families dominated Bacilli on day 0 (Figure 2C), and along with fermentation, their relative abundance dropped dramatically within a week, while Lactobacillaceae became prevailing till the end of fermentation (Supplementary Figure S3). Nevertheless, since the beginning of fermentation, the read numbers of ptb and buk significantly decreased, indicating Bacilli was no longer the major source of the butyrate producing key enzymes. It should be mentioned that a significant correlation (*r* = 0.812, *p* = 0.001) was found between dynamics of lactate, the major organic acid in fermented grains, and the succession of Bacilli in fermented grains. Moreover, among the three predominant classes in fermented grains, the correlation relationship between butyrate and Clostridia (*r* = 0.835, *p* = 0.019) was the closest, followed by Bacilli (*r* = 0.670, *p* = 0.012), while no significant correlation was identified for Bacteroidia (*r* = 0.099, *p* = 0.748). Since there is no record of real *but* from Bacilli in FunGene database^7^, the predictive atoD and atoA in Bacilli might represent other kinds of CoA transferases.

Compared with Bacilli, Clostridia (mean relative abundance = 2.21%), and Bacteroidia (mean relative abundance = 2.93%) were observed with rather exiguous abundance during the fermentation process (Figure 2B). Both the *buk* and *but* pathways were detected in Clostridia, while only the *buk* pathway was identified in Bacteroidia (Figure 2A). Presumptive Bacteroidia butyrate producers could only be detected in the fermented grains on days 7, 14, 20, and 30, and Prevotellaceae (order Bacteroidales) was the predominant butyrate-producing Bacteroidia community in the sample on day 7 (Figure 2D). Clostridia was identified as the potential butyrate producers throughout fermentation and dominated butyrate production in the samples on days 14, 20, 30, and 40 (Figure 2A). Furthermore, the hiatus of ACSM in all samples suggested that butyrate in fermented grains was not widely utilized as a carbon source by bacteria.

### Clone Library Analysis of *buk* and *but* Genes

**Figure 3:**
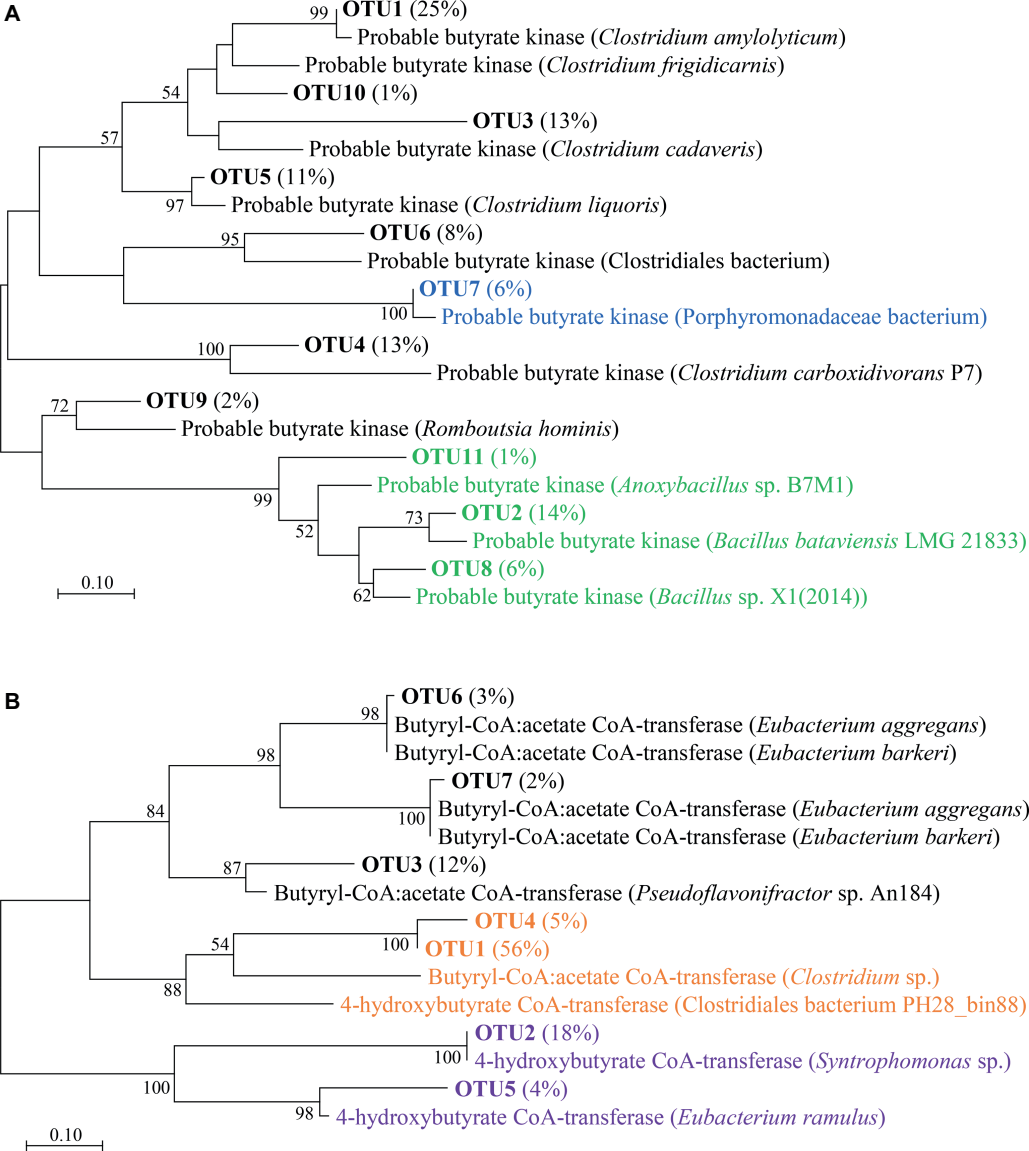
Phylogenetic diversities of *buk* and *but* genes in the fermented grains of Luzhou-flavor *baijiu*. **(A)** A neighbor-joining tree of deduced protein sequences of *buk* genes. Green, blue and black branches indicate Bacilli, Bacteroidia and Clostridia, respectively. **(B)** A neighbor-joining tree of deduced protein sequences of *but* genes. Black, orange and purple branches indicate real *but*, candidate *but* and 4-hydroxybutyrate CoA-transferase gene, respectively. For **(A)** and **(B)**, bootstrap values from 1,000 replicates are included. Percentage in the bracket shows the relative abundance of specific OTU.

In order to evaluate the predictive functional profiles of bacterial community and further investigate butyrate-producing bacteria in fermented grains, we performed clone library analysis based on the key-enzyme coding genes *buk* and *but* in butyrate synthesis pathway. A total of 100 clones from each clone library were assigned to 11 OTUs and seven OTUs, respectively (Figure 3).

As for the *buk* clone library, all the OTUs were confirmed as real *buk* sequences (Figure 3A). According to the BLASTx results, OTU2 (relative abundance = 14%), OTU8 (6%), and OTU11 (1%) fell into class Bacilli; OTU7 (6%) belonged to Bacteroidia; relative abundance of the left seven OTUs accounted for 73% of all identified *buk* clones that were scattered in Clostridia and five of them were further annotated as different species of genus *Clostridium* (Supplementary Table S3). With regard to the *but* gene (Figure 3B), due to its high sequence similarity to 4-hydroxybutyrate CoA-transferase (4-Hbt) (Charrier et al., 2006), which also participated in the conversion of butyryl-CoA to butyrate (Louis and Flint, 2017), we failed to exclude the amplification of these distinct genes. Only OTU3 (12%), OTU6 (3%), and OTU7 (2%) were confirmed as real *but* sequences. Since the subtle similarities to both *but* and 4-*Hbt* gene were detected, OTU1 (56%) and OTU4 (5%) were also considered as candidate *but* sequences (Supplementary Table S4). The closest reference sequences of all *but* OTUs belonged to Clostridia. Based on this part, Clostridia was considered as the potential major butyrate-producing bacteria in both *buk* and *but* pathways. Comprehensive considerations combined with functional prediction, we decided to investigate the Clostridial community *via* Clostridia-specific 16S rRNA gene sequencing analysis to further zoom in on the Clostridia with butyrate-producing potential in fermented grains.

### Taxonomic Diversity of Clostridia and their Butyrate Metabolic Profiling Prediction

**Figure 4:**
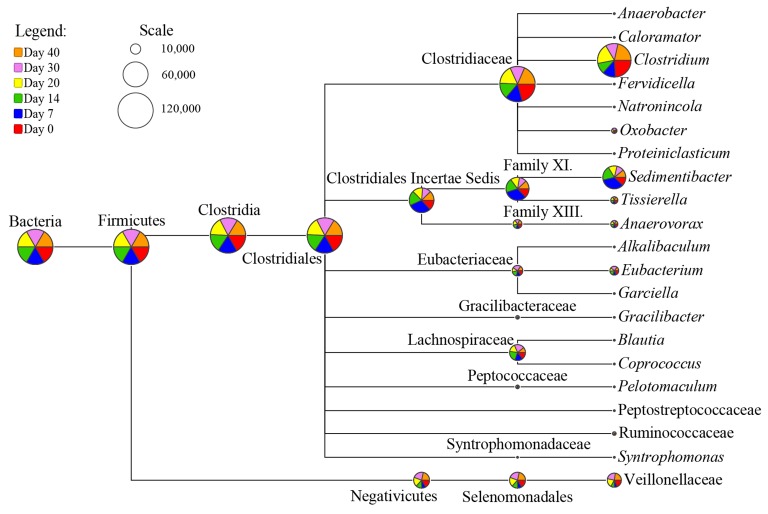
Dynamic patterns of Clostridial consortia in the fermented grains during *baijiu* fermentation. The results were based on Clostridia-specific 16S rRNA gene sequencing analysis. Scale represents read numbers. Area of each pie chart in the node of phylogenetic tree is proportional to the square root of reads assigned.

As Clostridia was not predominant in microbial community of fermented grains (Figure 2B), using universal bacterial primer sets for sequencing analysis possibly caused the loss of information about functioning Clostridia irrespective of their abundance. Therefore, Clostridial specific primer set SJ-F/SJ-R was applied for sequencing to interpret the taxonomic diversity of Clostridia. After quality-filtration, 20,000 reads per sample were clustered into 2,779 OTUs in total based on 3% dissimilarity of 16S rRNA gene sequences. Among them, 89.43% OTUs were affiliated with Clostridia, while the rest were assigned to class Negativicutes (further annotated as family Veillonellaceae), which formerly belonged to Clostridial cluster IX (Marchandin et al., 2010). At the order level, Clostridia was dominated by Clostridiales (98.72%), of which 29.23% remained unclassified at the family level. As for the taxonomic distribution at the genus level, 152 OTUs with the average relative abundance per sample >0.1% were selected for analysis. A total of 18 genera scattered in 10 families (Clostridiaceae, Clostridiales Incertae Sedis Family XI and Family XIII, Lachnospiraceae, Eubacteriaceae, Gracilibacteraceae, Peptococcaceae, Peptostreptococcaceae, Ruminococcaceae, and Syntrophomonadaceae) were identified in fermented grains throughout fermentation (Figure 4, Supplementary Figure S4). The relative abundance of *Clostridium* was the highest among the 18 identified genera, followed by *Sedimentibacter*. Subsequently, their potential metabolic features were analyzed *via* PICRUSt to further reveal Clostridial butyrate producers.

**Figure 5:**
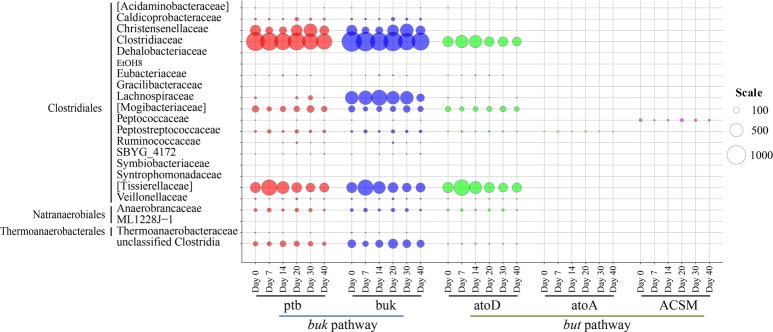
Family-level distribution of Clostridia in butyrate synthetic pathways during *baijiu* fermentation analyzed by Clostridia-specific 16S rRNA gene sequencing and PICRUSt analysis. The diameter of bubbles (scale) is proportional to the read numbers of enzymes.

The family-level distribution of potential Clostridia butyrate producers is displayed in Figure 5. In agreement with the PICRUSt analysis results of bacterial community, here *buk* pathway was also considered as the possibly major synthetic pathway of butyrate in fermented grains. Clostridiaceae (mean relative abundance = 38.12%) and [Tissierellaceae] (mean relative abundance = 12.30%) were the predominant contributors of butyrate synthesis key enzymes, including ptb, buk, and atoD. Family [Tissierellaceae] was classified in the Clostridiales Incertae Sedis Family XI with the RDP database. The *buk* pathway was mainly observed in Clostridiaceae, [Tissierellaceae], Christensenellaceae, Lachnospiraceae, and [Mogibacteriaceae], while the *but* pathway was merely identified in Clostridiaceae, [Tissierellaceae], and [Mogibacteriaceae]. Read numbers of these enzymes demonstrated different variation patterns along with fermentation. In general, read numbers of ptb, buk, and atoD in [Tissierellaceae] and atoD in Clostridiaceae were downregulated, while upregulation of ptb and buk was detected in Christensenellaceae (Figure 5). It is worth noting that read numbers of ptb and buk in Clostridiaceae almost remained constant during brewing, and ranked first compared with other families. As *Clostridium* dominated Clostridiaceae (Figure 4), we deduced that *Clostridium* might be the crucial butyrate producers in fermented grains based on the above-mentioned studies.

### Evaluation of *Clostridium* Butyrate-Producing Potential by Culture Method

According to the Clostridia-specific 16S rRNA gene sequences, the media were predicted for Clostridia by GROWREC in KOMODO database. In total, 16 kinds of media for *Clostridium* (media ID in DSMZ database: 1, 58, 78, 110, 104b, 136, 144, 156, 453, 514, 671, 830, 872, 898.3, 915.2, and 960.4; link: http://komodo.modelseed.org/servlet/KomodoTomcatServerSideUtilitiesModelSeed?MediaList) were predicted based on the 16S rRNA gene sequences of 42 OTUs belonging to genus *Clostridium*. A total of 17 *Clostridium* species were isolated from the fermented grains of Luzhou-flavor *baijiu* using the predicted media (Table 1).

**Table 1:** Profiles of short-chain fatty acids in the fermentation broth of *Clostridium* strains isolated with the media predicted by GROWREC in Known Media Database.

Strains	GenBank accession no.	Identity (%)	Media ID in the DSMZ database^b^	Butyrate synthesis pathways		Production of fatty acids (g/L)^c^			
				*buk*	*but*	Acetate	Butyrate	Pentanoate	Caproate
**Clostridium acetobutylicum**	KJ951058.1	100	156	−^a^	−	0.23 ± 0.00	ND^d^	ND	ND
*Clostridium aminovalericum*	NR_113199.1	99	104b	+	−	1.28 ± 0.11	6.70 ± 0.47	1.43 ± 0.06	3.20 ± 0.21
*Clostridium amylolyticum*	NR_044386.1	99	136	+	−	0.18 ± 0.47	7.71 ± 0.52	ND	0.33 ± 0.05
**Clostridium botulinum**	CP013243.1	100	104b	+	−	0.93 ± 0.06	ND	1.89 ± 0.08	2.63 ± 0.13
*Clostridium butyricum*	HQ328064	100	1	+	−	1.22 ± 0.13	1.52 ± 0.05	0.03 ± 0.01	ND
*Clostridium carboxidivorans*	CP011803.1	99	156	+	−	1.49 ± 0.08	ND	0.36 ± 0.08	0.49 ± 0.07
*Clostridium kluyveri*	NR_074447.1	100	220	−	+	ND	0.34 ± 0.05	ND	1.65 ± 0.23
**Clostridium kogasensis**	NR_136452.1	99	110	+	+	1.82 ± 0.08	0.54 ± 0.02	1.34 ± 0.13	0.79 ± 0.11
*Clostridium luticellarii*	KP342257.1	99	220	−	+	ND	0.13 ± 0.02	ND	ND
**Clostridium mangenotii**	NR_104729.1	100	1	+	−	ND	0.09 ± 0.01	ND	ND
*Clostridium novyi*	LC193834.1	99	156	−	+	0.70 ± 0.66	5.77 ± 0.39	1.93 ± 0.09	2.80 ± 0.11
*Clostridium senegalense*	NR_125591.1	100	58	−	−	1.07 ± 0.02	ND	2.17 ± 0.10	3.03 ± 0.18
*Clostridium sporogenes*	NR_029231.1	100	104b	+	−	0.35 ± 0.06	0.28 ± 0.04	ND	3.20 ± 0.21
*Clostridium swellfunianum*	NR_126179.1	97	104b	−	−	0.88 ± 0.05	ND	ND	ND
*Clostridium tyrobutyricum*	CP014170.1	100	104b	−	+	1.05 ± 0.08	0.43 ± 0.06	0.64 ± 0.03	ND
*Clostridium* sp. ArC5	AF443594.1	99	104b	+	−	0.85 ± 0.00	4.00 ± 0.22	2.13 ± 0.11	2.84 ± 0.14
**Clostridium sp. YN5**	AB537983.1	97	104b	+	−	ND	0.25 ± 0.03	ND	1.53 ± 0.11

*Species highlighted by bold fonts were first isolated from *baijiu* brewing ecosystem according to Zou et al. (2018a) and Chai et al. (2019)*.

a No visible band around correct size.

b 1, nutrient agar; 58, Bifidobacterium medium; 104b, PYX medium; 110, chopped meat medium with carbohydrates; 136, Veillonella medium; 156, *Clostridium propionicum* medium; 220, CASO Agar (Merck 105458); 520, *Clostridium cellulolyticum* (CM3). According to the medium number, the detailed medium formulation can be found from the following link: http://komodo.modelseed.org/servlet/KomodoTomcatServerSideUtilitiesModelSeed?MediaList

c *Values are expressed as means ± standard deviation*.

d *Not detected or the peak area is over 100-fold smaller than internal standard*.

After specific isolation, all *Clostridium* strains were cultured in the Clostridial universal medium RCM to assess and compare their butyrate-producing capacities. Among the 17 species of *Clostridium*, 12 species (*C. aminovalericum, C. amylolyticum, C. butyricum, C. kluyveri, C. kogasensis, C. luticellarii, C. mangenotii, C. novyi, C. sporogenes, C. tyrobutyricum, C.* sp. ArC5, and *C.* sp. YN5) could produce butyrate, wherein the butyrate production of *C. amylolyticum* was the highest (7.71 g/L), followed by *C. aminovalericum* (6.70 g/L), *C. novyi* (5.77 g/L), and *C.* sp. ArC5 (4.00 g/L) (Table 1). Here, *Clostridium amylolyticum* (Song and Dong, 2008), *C. mangenotii* (Lawson et al., 2016), and *C.* sp. ArC5 were first identified with butyrate-producing potential, possibly due to different culture conditions. With regard to the butyrate synthesis genes, *Clostridium aminovalericum, C. amylolyticum, C. butyricum, C. mangenotii, C. sporogenes, C.* sp. ArC5, and *C.* sp. YN5 were found to have *buk* gene by PCR amplification, while *but* gene could be amplified in *C. kluyveri, C. luticellarii, C. novyi*, and *C. tyrobutyricum. Clostridium kogasensis* was the only one species possessing both *buk* and *but* genes.

## Discussion

After undergoing a multispecies solid-state fermentation process, Chinese *baijiu* is finally produced *via* distillation. Among the microbial metabolites, butyrate and its derivatives are important aroma compounds in Luzhou-flavor *baijiu* (Fan and Qian, 2006). Hence, studying the butyrate producers during the fermentation process could help to develop a deeper understanding of the fermentative mechanisms of Luzhou-flavor *baijiu* and locate the key functional community contributing to the formation of its unique flavor. Though anaerobic bacteria, especially *Clostridium*, were considered as important butyrate producers in the pit mud of *baijiu* during the sealed-in-cellar manufacturing process (Hu et al., 2015; Liu et al., 2015), there was no report specifically elucidating butyrate-producing bacterial assembly in the fermentation grains of *baijiu* during brewing.

In this study, PICRUSt analysis based on the bacterial 16S rRNA gene sequences revealed that the *buk* pathway could be the major butyrate biosynthesis pathway in fermented grains of Luzhou-flavor *baijiu* (Figure 2A). The *buk* gene was usually more abundant in the intestine of many carnivores, and the lack of consistent acetate supply was considered as the major factor that limit the performance of *but* (Vital et al., 2015). However, as the fermentation matrix, the fermented grains consisted entirely of sorghum and other grains; the relative high content of acetate in the fermented grains (around 1.40 g/kg) indicated that there might be other factors influencing the distribution of the two pathways in different environments.

At the class level, Bacilli, Clostridia, and Bacteroidia were found as three main potential butyrate-producing groups in fermented grains (Figure 2). Bacilli and Bacteroidia possessed the *buk* pathway, while Clostridia was able to produce butyrate *via* both the *buk* and *but* pathways. Bacillaceae and Staphylococcaceae belonging to Bacilli were the major potential contributors of butyrate kinase at the beginning of fermentation, which was also considered as associated with butyrate production in human gut (Magnúsdóttir et al., 2017). However, along with the fermentation, lactic acid bacteria, especially Lactobacillaceae became dominant, and possibly inhibited the proliferation of Bacillaceae and Staphylococcaceae by producing lactate. It should be mentioned that butyrate producers from Clostridia and Bacteroidia were not significantly affected by lactate, which related to their ability of utilizing lactate as a carbon source (Reichardt et al., 2014). Moreover, the spatial heterogeneity characterized the solid-state fermentation environment of baijiu, which could generate numerous ecological niches with divergent environmental factors. This was also conductive to explain the negligible impact of lactate on Clostridia and Bacteroidia. Prevotellaceae was the major Bacteroidia butyrate-producing group in fermented grains (especially on day 7). Nevertheless, Bacteroidia was found mainly composed by *Bacteroides* (Bacteroidaceae) and regarded as non-butyrate producer (Reichardt et al., 2014).

The *buk* and *but* pathways that directly involved in bacterial butyrate synthesis in fermented grains were further examined by clone library analysis of *but* and *buk* partial gene amplicons. The sequences of the *buk* gene mainly belonged to Clostridia, followed by Bacilli and Bacteroidia, while those of the *but* gene belonged to Clostridia (Figure 3). Due to the low identity to reference sequences (<80%), some OTUs in these two libraries could not be annotated at the genus or species level. As the information in the reference database is improved, the taxonomic and functional assignment of sequencing reads in this study will be more accurate.

Similar to pit mud, Clostridia were also regarded as the major potential butyrate-producing bacteria in fermented grains (Chai et al., 2019). However, it was not widely studied in previous studies of fermented grains due to its low relative abundance as compared with lactic acid bacteria (Zou et al., 2018b). Thus, diversity and succession of Clostridia in the fermented grains of Luzhou-flavor *baijiu* was systematically studied by Clostridia-specific 16S rRNA gene sequencing. The majority of Clostridia belong to Clostridiales, in which Clostridiaceae and Clostridiales Incertae Sedis Family XI ([Tissierellaceae]) are the two dominant families. *Clostridium* (Clostridiaceae) was predicted as a major potential butyrate producer in the fermented grains (Figure 4). It stands to reason that endpoint metagenomes just tell us which organisms have been present at some point in the process but may have died and left their DNA in the actual sample. As such, unraveling the dynamic expression of genes in *baijiu* microbiota at transcriptional and protein level is ongoing in further studies, e.g., RNA-based DGGE and 16S rRNA gene sequencing approaches have been used in elucidating functional microbes in food fermentation microbial ecosystem (Alessandria et al., 2016; Wuyts et al., 2018). Reinforced Clostridial Medium (RCM) and ethanol/sodium acetate (ES) medium were used to isolate various *Clostridium* strains from pit mud in the previous work (Chai et al., 2019). However, different from pit mud, *Clostridium* assembly was a tiny minority among bacteria in fermented grains, and merely four species of *Clostridium* were isolated from the fermented grains by RCM and ES media in our preliminary study (data not shown). To convince the results from molecular approach, a 16S rRNA gene sequence-guided culture medium prediction method was applied to isolate the *Clostridium* strains. A total of 16 media were successfully predicted by GROWREC in KOMODO database, which facilitated the isolation of strains belonging to 17 *Clostridium* species from the fermented grains (Table1). Five species (*C. acetobutylicum, C. botulinum, C. kogasensis, C. mangenotii*, and *C.* sp. YN5) have not been reported in the fermentation of *baijiu* in previous studies (Zou et al., 2018a; Chai et al., 2019). Among the 17 species, 12 species of *Clostridium* could produce butyrate in the fermentation broth *via* the *buk* and/or *but* pathways (Table 1), which validated the phylogenetic and functional diversities of butyrate producer in the fermented grains of *baijiu*. However, in pit mud, *buk* pathway might dominate the butyrate production of *Clostridium via* metagenomic analysis (Tao et al., 2017). The coupled metagenomics and GROWREC method might be used in future study for isolating other potential butyrate producers (even with low abundance) in the fermented grains, such as *Sedimentibacter* species.

In summary, this study revealed that Clostridia functioned as an important functional group driving the butyrate formation *via* the *buk* and *but* pathways by clone library analysis of *but* and *buk* partial gene amplicons, high throughput sequencing and PICRUSt functional analysis of 16S rRNA genes. The genus *Clostridium* was found as the major potential butyrate producer among 18 genera in the class Clostridia by Clostridia-specific 16S rRNA gene sequencing and PICRUSt analysis. A total of 17 species of *Clostridium* were successfully isolated by the GROWREC-aided method, and the profiles of fatty acids and functional genes (*but* and *buk*) in these species validated the phylogenetic and functional diversities of butyrate-producing bacteria in the *baijiu* microbiota. We plan to further elucidating the similarities and differences of bacterial butyrate-synthesizing mechanisms between pit mud and fermented grains during brewing.

## Data Availability

No datasets were generated or analyzed for this study.

## Author Contributions

Z-ML and Z-HX designed the experiments. JM, L-JC, P-XX, WQ, and CX conducted the experiments and analyzed the results. L-JC, Z-ML, and X-JZ prepared the manuscript. S-TW, C-HS, and J-SS assisted in drafting the manuscript. All authors read and approved the final manuscript.

### Conflict of Interest Statement

The authors declare that the research was conducted in the absence of any commercial or financial relationships that could be construed as a potential conflict of interest.

## Supplementary Material

The Supplementary Material for this article can be found online at: https://www.frontiersin.org/articles/10.3389/fmicb.2019.01397/full#supplementary-material

## Footnotes

^1^https://4virology.net/virology-ca-tools/j-codehop/

^2^http://www.drive5.com/uparse/

^3^http://rdp.cme.msu.edu/classifier/classifier.jsp

^4^http://huttenhower.sph.harvard.edu/galaxy

^5^http://fungene.cme.msu.edu/FunGenePipeline/

^6^http://www.uniprot.org/blast/

^7^http://fungene.cme.msu.edu/index.spr
