# Supplementary material for: Zooming in on Butyrate-Producing Clostridial Consortia in the Fermented Grains of Baijiu via Gene Sequence-Guided Microbial Isolation
Source: Front Microbiol. 2019 Jun 21;10:1397. doi: 10.3389/fmicb.2019.01397 (PMC6611424; doi:10.3389/fmicb.2019.01397)
Supplement: Supplementary file 1 [file Data_Sheet_1.docx]

Frontiers in Microbiology

Supplementary data for the manuscript:

**Zooming in on butyrate-producing Clostridial consortia in the fermented grains of baijiu via gene sequence-guided microbial isolation**

Li-Juan Chai^1,4^, Zhen-Ming Lu^1,4,5^, Xiao-Juan Zhang^1,4,5^, Jian Ma^2^, Peng-Xiang Xu^2^, Wei Qian^2^, Chen Xiao^2^, Song-Tao Wang^5^, Cai-Hong Shen^5^, Jin-Song Shi^3^ and Zheng-Hong Xu^1,2,5,*^

^1^National Engineering Laboratory for Cereal Fermentation Technology, Jiangnan University, Wuxi 214122, P.R. China;

^2^Key Laboratory of Industrial Biotechnology, Ministry of Education, Jiangnan University, Wuxi 214122, P.R. China;

^3^School of Pharmaceutical Science, Jiangnan University, Wuxi 214122, P.R. China;

^4^Jiangsu Engineering Research Center for Bioactive Products Processing Technology, Jiangnan University, Wuxi 214122, P.R. China;

^5^National Engineering Research Center of Solid-State Brewing, Luzhou 646000, P.R. China;

*Corresponding author: National Engineering Laboratory for Cereal Fermentation Technology, Jiangnan University, 1800 Lihu Avenue, Wuxi 214122, P.R. China; E-mail: zhenghxu@jiangnan.edu.cn; Tel.: +86-510-85918206; Fax: +86-510-85918206.

**Supplementary figure legends**

**Supplementary Fig. 1** Photos of Luzhou-flavor *baijiu* fermentation pit. Fermentation pit was covered with polyethylene cloth to keep moist during brewing.

**Supplementary Fig. 2** Dynamics of physiochemical parameters in fermented grains during fermentation process. (A) Temperature; (B) Ethanol; (C) Moisture; (D) pH; (E) Lactate content.

**Supplementary Fig. 3** Dynamics of Bacillaceae, Staphylococcaceae and Lactobacillaceae families, belonging to class Bacilli, in fermented grains during fermentation process.

**Supplementary Fig. 4** Succession of the top 18 genera in fermented grains during fermentation process according to Clostridial specific-16S rRNA gene sequencing analysis.

**Supplementary Fig. 1**


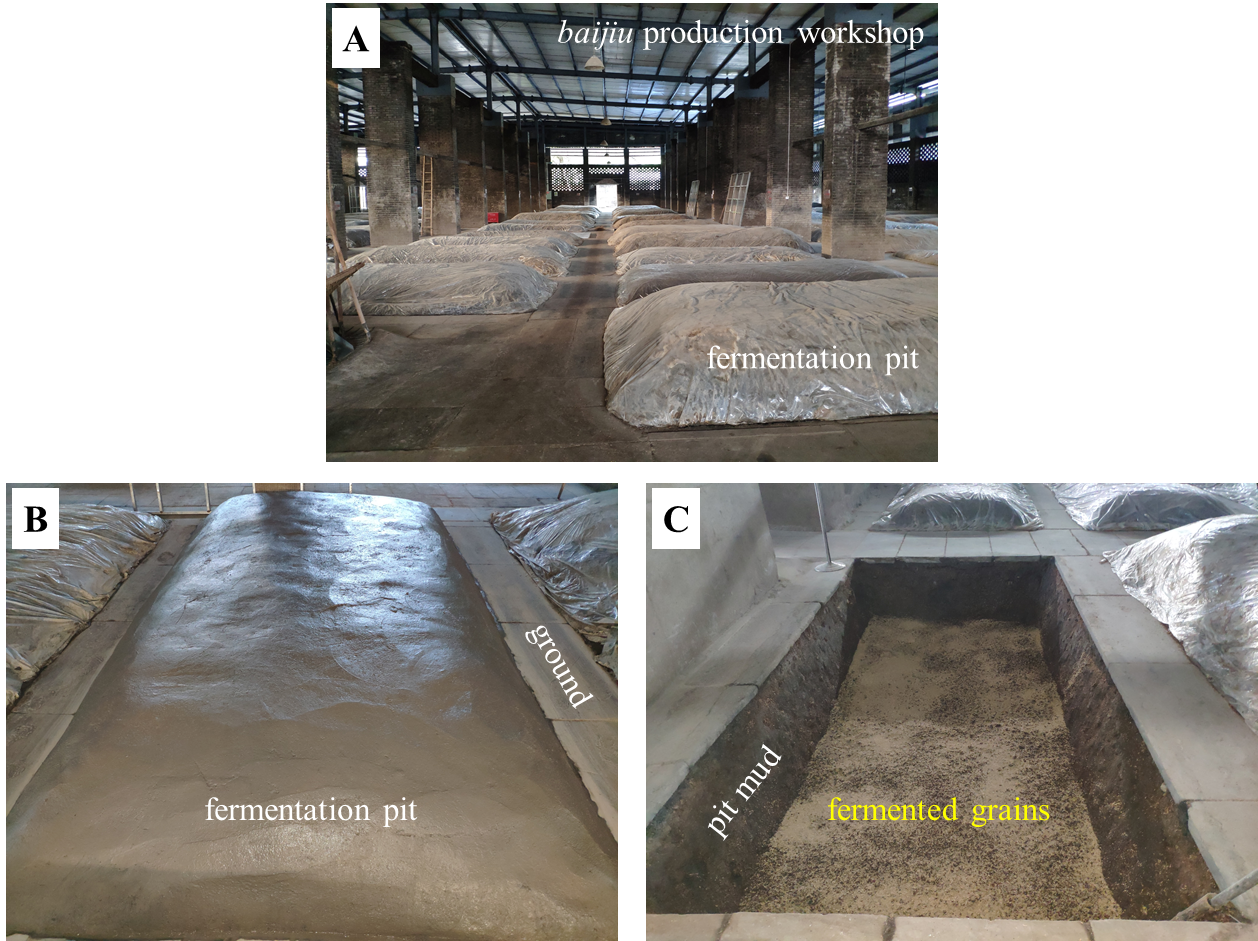


**Supplementary Fig. 2**


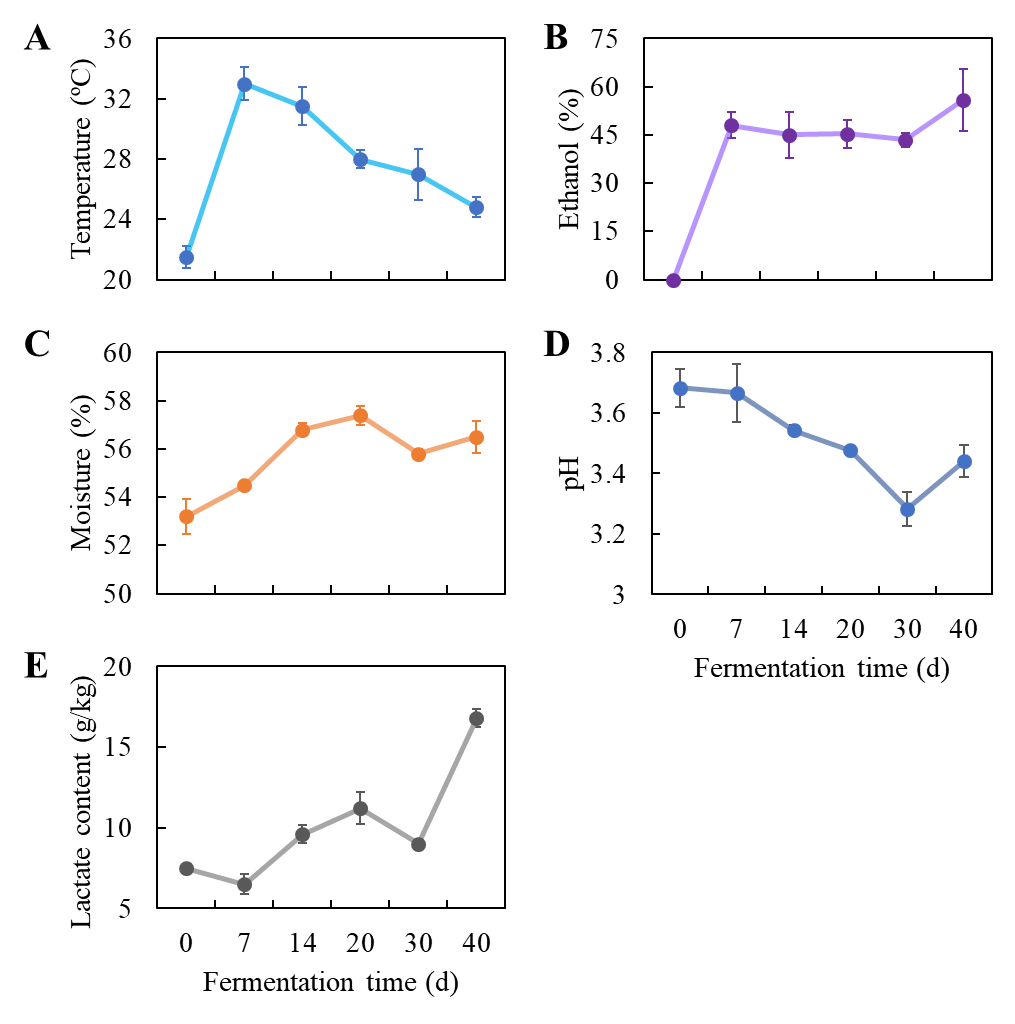


**Supplementary Fig. 3**


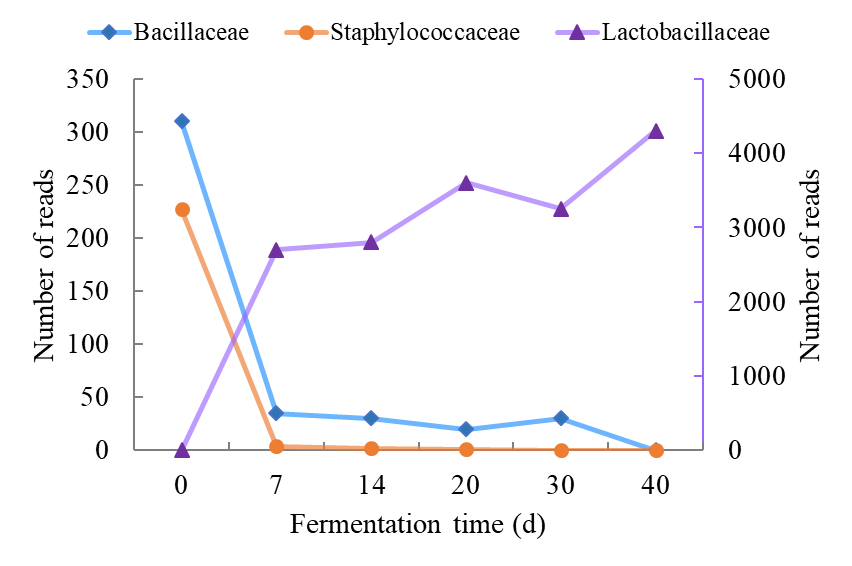


**Supplementary Fig. 4**


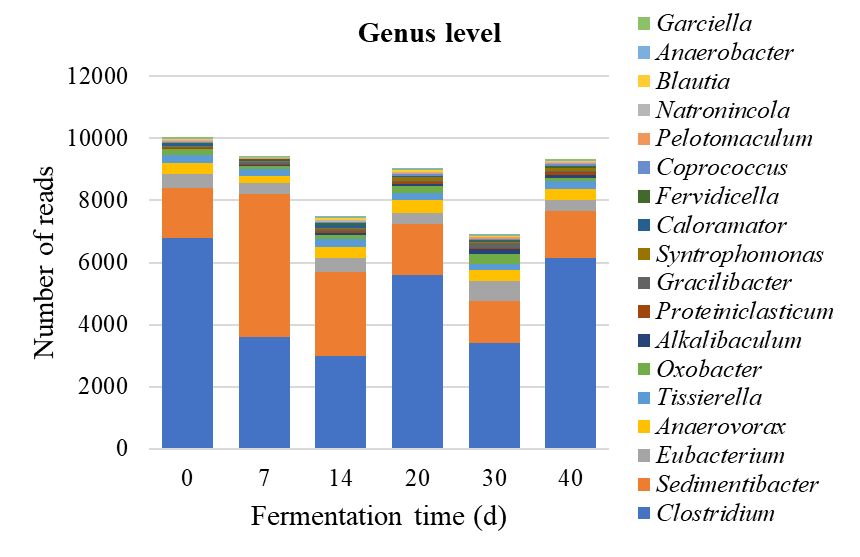


**Supplementary Table 1** Primers used in this study

| Primer pairs | Sequence (5’-3’) | Amplicon size (kb) | Annealing temp (°C) | Reference |
| --- | --- | --- | --- | --- |
| *but*-F/*but*-R | ATGGGAACTAAGAANHTNTAYGA | 0.45 | 49.0 | Chai *et al.* (2019) |
|  | TAAATTTACCATTCCRWAYTCNGT |  |  |  |
| *buk*-F/*buk*-R | AAAATATTAATNATHAAYCCNGG | 0.50 | 47.0 | Chai *et al.* (2019) |
|  | GCCATTGCTTTTTGATTNARNRCRTG |  |  |  |
| P1/P2 | TGGAGAGTTTGATCCTGGCTCAG | 0.40 | 55.0 | Huang *et al.* (2011) |
|  | TACCGCGGCTGCTGGCAC |  |  |  |
| SJ-F/SJ-R | CGGTGAAATGCGTAGAKATTA | 0.27 | 56.5 | Hu *et al.* (2014) |
|  | CGAATTAAACCACATGCTCCG |  |  |  |
| 27F/1492R | AGAGTTTGATCCTGGCTCAG | 1.47 | 56.0 | Zhang *et al.* (2014) |
|  | GGTTACCTTGTTACGACTT |  |  |  |

The following sequences are targeted: *buk*-F/*buk*-R, butyrate kinase (*buk*) gene; *but*-F/*but*-R, butyryl-CoA:acetate CoA-transferase (*but*) gene; P1/P2, V1/V3 hypervariable region of 16S rRNA gene; SJ-F/SJ-R, V4/V5 hypervariable region of 16S rRNA gene in Clostridia; 27F/1492R, 16S rRNA gene.

**Supplementary Table 2** The basic sequencing information and alpha diversity indices of bacterial and Clostridial communities in fermented grains during brewing

| Days | Shannon | Inverse Simpson | Chao1 | Ace | Coverage |
| --- | --- | --- | --- | --- | --- |
| Bacterial 16S rRNA gene amplicon sequencing | | | | | |
| 0 | 5.10 | 0.864 | 1079.0 | 1076.6 | 98.8% |
| 7 | 5.86 | 0.960 | 641.0 | 648.3 | 99.4% |
| 14 | 4.89 | 0.865 | 780.1 | 741.4 | 99.2% |
| 20 | 4.71 | 0.867 | 684.1 | 703.4 | 99.3% |
| 30 | 5.99 | 0.924 | 1129.0 | 1095.0 | 98.8% |
| 40 | 5.89 | 0.931 | 909.4 | 880.1 | 99.2% |
| Clostridial specific-16S rRNA gene amplicon sequencing | | | | | |
| 0 | 6.03 | 0.954 | 2215.9 | 2292.0 | 96.3% |
| 7 | 5.72 | 0.902 | 2583.3 | 2727.1 | 88.5% |
| 14 | 5.39 | 0.853 | 2733.5 | 2839.5 | 98.7% |
| 20 | 5.85 | 0.914 | 1930.6 | 1945.2 | 84.2% |
| 30 | 6.52 | 0.943 | 2842.7 | 3000.4 | 90.7% |
| 40 | 6.35 | 0.928 | 2793.7 | 2849.3 | 88.2% |

**Supplementary Table 3** Clone library analysis of butyrate kinase (*buk*) gene

| OTUs | Closest reference sequence | UniProtKB accession no. | Identity (%) | Query coverage (%) | E-value | Relative abundance (%) |
| --- | --- | --- | --- | --- | --- | --- |
| OTU1 | Probable butyrate kinase (*Clostridium amylolyticum*) | A0A1M6HIY8 ﻿ | 98.1 | 99.0 | 4.5e-63 | 25 |
| OTU2 | Probable butyrate kinase (*Bacillus bataviensis* LMG 21833) | K6DN05 | 92.4 | 95.2 | 3.1e-60 | 14 |
| OTU3 | Probable butyrate kinase (*Clostridium cadaveris*) | A0A1I2L2S3 | 66.7 | 79.0 | 1.5e-39 | 13 |
| OTU4 | Probable butyrate kinase (*Clostridium carboxidivorans* P7) | C6PUK0 | 70.5 | 84.8 | 3.9e-43 | 13 |
| OTU5 | Probable butyrate kinase (*Clostridium liquoris*) | A0A2T0B9I2 | 94.3 | 97.1 | 1.2e-60 | 11 |
| OTU6 | Probable butyrate kinase (Clostridiales bacterium) | A0A352IHE4 | 74.8 | 89.3 | 1.4e-46 | 8 |
| OTU7 | Probable butyrate kinase (Porphyromonadaceae bacterium) | A0A355CA62 | 97.1 | 99.0 | 2.2e-61 | 6 |
| OTU8 | Probable butyrate kinase (*Bacillus* sp. X1(2014)) | A0A077J499 | 89.5 | 96.2 | 2.0e-58 | 6 |
| OTU9 | Probable butyrate kinase (*Romboutsia hominis*) | A0A2P2BV17 | 81.9 | 86.7 | 3.6e-49 | 2 |
| OTU10 | Probable butyrate kinase (*Clostridium frigidicarnis*) | A0A1I0Z541 | 83.8 | 92.4 | 7.6e-53 | 1 |
| OTU11 | Probable butyrate kinase (*Anoxybacillus* sp. B7M1) | A0A160FBD9 | 77.1 | 86.7 | 6.4e-48 | 1 |

**Supplementary Table 4** Clone library analysis of butyryl-CoA: acetate CoA-transferase (*but*) gene

| OTUs | Closest reference sequence | UniProtKB accession no. | Identity (%) | Query coverage (%) | E-value | Relative abundance (%) |
| --- | --- | --- | --- | --- | --- | --- |
| OTU1 | Butyryl-CoA:acetate CoA-transferase (*Clostridium* sp.) | A0A353J1E1 | 61.5 | 78.7 | 1.8e-46 | 56 |
| OTU2 | 4-hydroxybutyrate CoA-transferase (*Syntrophomonas* sp.) | A0A3D1QC51 | 97.5 | 98.3 | 9.2e-73 | 18 |
| OTU3 | Butyryl-CoA:acetate CoA-transferase (*Pseudoflavonifractor* sp. An184) | A0A1Y4L1X1 | 85.2 | 94.3 | 2.3e-68 | 12 |
| OTU4 | 4-hydroxybutyrate CoA-transferase (Clostridiales bacterium PH28_bin88) | A0A0M2U2G0 | 60.0 | 77.4 | 2e-40 | 5 |
| OTU5 | 4-hydroxybutyrate CoA-transferase (*Eubacterium ramulus*) | A0A2V1JT81 | 82.4 | 94.1 | 2.9e-63 | 4 |
| OTU6 | Butyryl-CoA:acetate CoA-transferase (*Eubacterium aggregans*) | A0A1H3Z6V0 | 99.1 | 99.1 | 5.4e-73 | 3 |
|  | Butyryl-CoA:acetate CoA-transferase (*Eubacterium barkeri* (*Clostridium barkeri*)) | A0A1H3I8V0 | 99.1 | 99.1 | 5.4e-73 |  |
| OTU7 | Butyryl-CoA:acetate CoA-transferase (*Eubacterium aggregans*) | A0A1H4AF70 | 96.7 | 99.2 | 3.7e-77 | 2 |
|  | Butyryl-CoA:acetate CoA-transferase (*Eubacterium barkeri* (*Clostridium barkeri*)) | A0A1H3AUQ8 | 96.7 | 99.2 | 3.7e-77 |  |
